# Microbiota Alterations in Patients with Autoimmune Thyroid Diseases: A Systematic Review

**DOI:** 10.3390/ijms232113450

**Published:** 2022-11-03

**Authors:** Nadia Sawicka-Gutaj, Dawid Gruszczyński, Natalia Zawalna, Kacper Nijakowski, Ilaria Muller, Tomasz Karpiński, Mario Salvi, Marek Ruchała

**Affiliations:** 1 Department of Endocrinology, Metabolism and Internal Medicine, Poznan University of Medical Sciences, 60-355 Poznan, Poland; 2 Department of Conservative Dentistry and Endodontics, Poznan University of Medical Sciences, 60-812 Poznan, Poland; 3 Department of Endocrinology, Fondazione IRCCS Cà Granda, Ospedale Maggiore Policlinico, 20122 Milan, Italy; 4 Graves’ Orbitopathy Center, Fondazione IRCCS Cà Granda, Ospedale Maggiore Policlinico, 20122 Milan, Italy; 5 Department of Clinical Sciences and Community Health, University of Milan, 20122 Milan, Italy; 6 Department of Medical Microbiology, Poznan University of Medical Sciences, 61-712 Poznan, Poland

**Keywords:** autoimmune thyroid disease, Graves’ disease, Hashimoto’s thyroiditis, Graves’ orbitopathy, microbiota, microbiome, microbes

## Abstract

Autoimmune thyroid diseases (AITDs) are chronic autoimmune disorders that cause impaired immunoregulation, leading to specific immune responses against thyroid antigens. Graves’ disease (GD) and Hashimoto’s thyroiditis (HT) are the major forms of AITDs. Increasing evidence suggests a possible role of microbiota alterations in the pathogenesis and progression of AITDs. This systematic review was designed to address the following question: “Is microbiota altered in patients with AITDs?” After screening the selected studies using the inclusion and exclusion criteria, 16 studies were included in this review (in accordance with PRISMA statement guidelines). A meta-analysis revealed that patients with HT showed significantly higher values of diversity indices (except for the Simpson index) and that patients with GD showed significant tendencies toward lower values of all assessed indices compared with healthy subjects. However, the latter demonstrated a higher relative abundance of *Bacteroidetes* and *Actinobacteria* at the phylum level and thus *Prevotella* and *Bifidobacterium* at the genus level, respectively. Thyroid peroxidase antibodies showed the most significant positive and negative correlations between bacterial levels and thyroid functional parameters. In conclusion, significant alterations in the diversity and composition of the intestinal microbiota were observed in both GD and HT patients.

## 1. Introduction

Autoimmune thyroid diseases (AITDs) are the most common autoimmune diseases, manifesting primarily in the forms of Hashimoto’s thyroiditis (HT) and Graves’ disease (GD) [1]. Their pathogenesis refers to genetic predisposition, environmental factors, and disturbances in the functioning of the immune system [2]. Immune disorders lead to reactivity to thyroid autoantigens such as thyroid peroxidase (TPO), thyroglobulin (TG), and thyroid-stimulating hormone receptor. This causes an inflammatory infiltration of the thyroid gland and the production of cytokines, which impact the cells of the immune system and the follicle cells of the thyroid [1,3]. Despite sharing a common autoimmune cause, HT and GD show contrasting effects on thyroid function and thus contrasting clinical symptoms: HT, which determines hypothyroidism, is associated with weight gain, fatigue, weakness, dry skin [4], anemia [5], and predisposition to depressive conditions [6], even if euthyroidism is present, whereas GD, the most common cause of hyperthyroidism in iodine-sufficient areas, co-occurs with weight loss, heat intolerance, trembling, anxiety, tachycardia, and irritability [7]. Graves’ ophthalmopathy (GO) is a characteristic complication of GD, which in mild form affects 25–50% of patients with GD and manifests as eye pain, eyelid edema, excessive tearing, and light sensitivity. However, a minority of patients experience vision deterioration, corneal breakdown, and optic nerve neuropathy [8,9].

Recently, the role of microbiota in autoimmune diseases has gained much attention. Every human being has a unique composition of intestinal microbes, and there is no single optimal pattern. However, in most of the cases, *Firmicutes* and *Bacteroidetes* are the predominant phyla. The composition of the microbiome is variable and depends on several factors such as gender, age, lifestyle, physical activity, drug therapies, and diet [10,11].

Studies have reported the association between dysbiosis and the onset of diseases such as type 1 diabetes, rheumatoid arthritis, inflammatory bowel disease, systemic lupus erythematosus, autoimmune dermatitis, and autoimmune neurological diseases [12]. In these diseases, interactions in the pathomechanism can be observed at the level of microbes and their metabolites. Several pathomechanisms focusing on AITDs are available in the literature. Firstly, alterations in the composition of intestinal bacteria can lead to increased intestinal permeability [13], which is associated with an increased level of zonulin, a protein responsible for the regulation of intercellular connection [14,15]. The decreased tightness of enterocytes enables the penetration of microbiome antigens and the activation of the immune system via molecular mimicry [16]. Some bacterial antigens in the intestine have structures similar to those of autoantigens. Due to this analogy, plasma cells may be activated to generate antibodies that bind to antigens expressed on thyroid follicle cells and orbital fibroblasts in GO [17]. Dysbiosis also results in an increased production of autoantibodies by posttranslational protein modification. Furthermore, it contributes to the development of AITDs by shifting the Th1 helper lymphocyte pool to Th2 and inducing Toll-like receptor 4’s activation [18,19].

Similarly, metabolites of the microbiome significantly affect thyroid function. Research studies have focused primarily on short-chain fatty acids (SCFAs), which strengthen the integrity of enterocytes, protect against the intrusion of pathological microbes, modulate the immune system, and inhibit inflammatory processes [20,21]. In addition, SCFAs are primarily involved in the modulation of the balance between Th17 and Treg populations, which are closely related to the development of autoimmune diseases [17,22].

**Figure 1:**
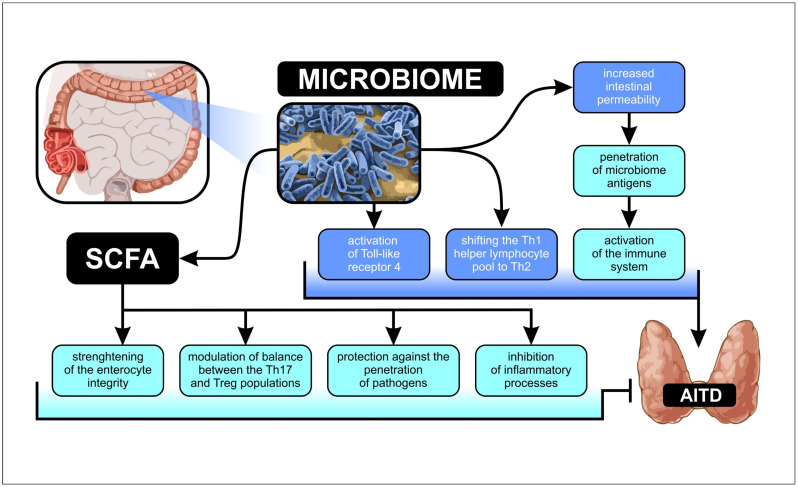
Potential relationships between alterations in gut microbiota and autoimmune thyroid disease pathogenesis. (AITD, autoimmune thyroid disease; SCFA, short-chain fatty acid).

Furthermore, the gut–thyroid axis includes the involvement of the microbiome in the metabolism of thyroid hormones. Previous studies have indicated the presence of deiodinases in the human intestine. Studies using animal models have reported the ability of intestinal bacteria to absorb deconjugated iodothyronine and even the competition to bind thyroid hormones to albumin [13,23]. In addition, some studies have reported that intestinal microbes contribute to the enterohepatic metabolism of thyroid hormones [24,25]. Furthermore, the microbiota influences the uptake of microelements necessary for the functioning of the thyroid gland, such as iodine, copper, iron, selenium, and zinc. Animal models have shown limited iodine uptake in individuals lacking the microbiome; however, no such association has been observed in parenterally fed humans with short bowel syndrome or after bariatric surgery [20,26,27]. In contrast, the competitive bacterial uptake of selenium has been observed, thereby reducing the bioavailability under reduced selenium conditions [26,28,29]. The diversity of reports on the interaction between the microbiota and the thyroid gland prompts an investigation into the involvement of the microbiome in thyroid disorders (Figure 1).

This systematic review was designed to answer the following question: “Is microbiota altered in patients with autoimmune thyroid diseases?”, which was formulated according to PICO (“Population”, “Intervention”, “Comparison” and “Outcome”).

## 2. Results

**Figure 2:**
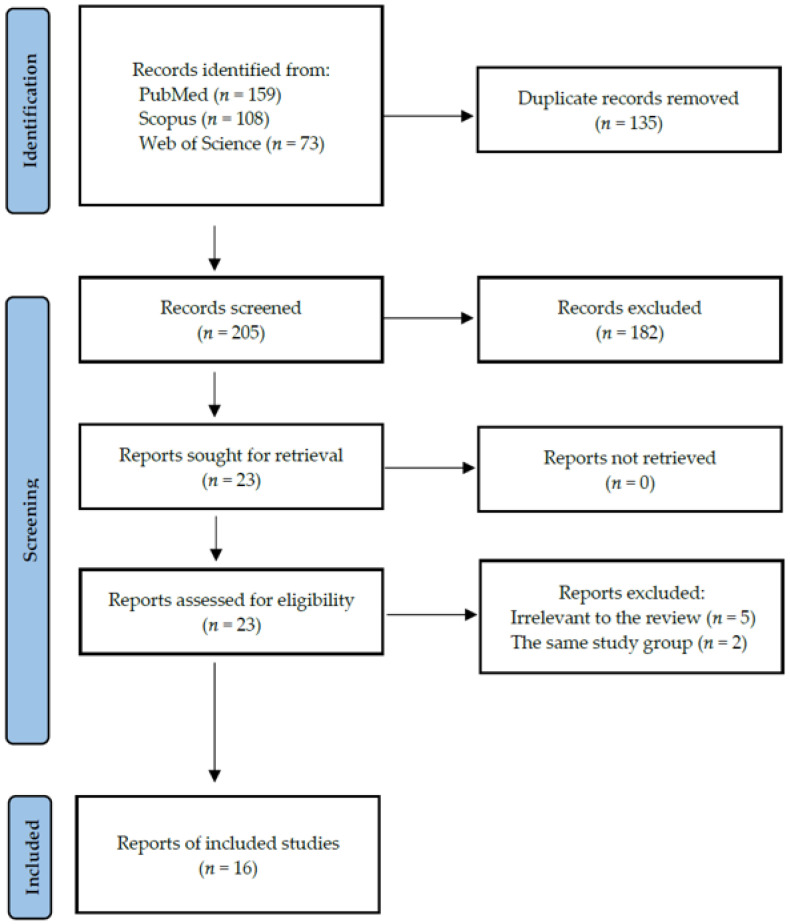
PRISMA flow diagram representing the search strategy.

After screening the studies using the inclusion and exclusion criteria, 16 studies were included in this review, thus including data collected in five different countries from a total of 761 human participants with diagnosed AITDs (including 563 with GD and 198 with HT) and 488 controls. The detailed selection strategy of the studies is shown in Figure 2. The inclusion and exclusion criteria are presented in Section 4.

From each eligible study included in this systematic review, data on general characteristics were collected, such as the year of publication and setting, participants, AITD diagnosis, inclusion and exclusion criteria, thyroid parameters determined, and medications supplemented (Table 1). The detailed characteristics are presented in Table 2, such as the type of laboratory material, methods of microbiological analysis, altered microbiota composition (at the phylum and genus levels), and changes in richness (ACE and Chao1) and diversity (Simpson and Shannon) indices. All studies examined fecal samples, which were analyzed using 16S rRNA gene sequencing (except for one study that used 16S rDNA gene sequencing).

**Figure 3:**
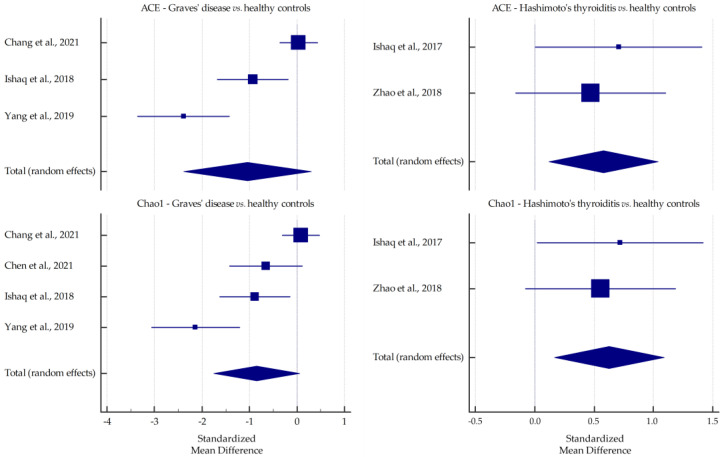
Pooled standardized mean differences in ACE and Chao1, shown separately for Graves’ disease and Hashimoto’s thyroiditis [30,31,32,39,43,45].

**Figure 4:**
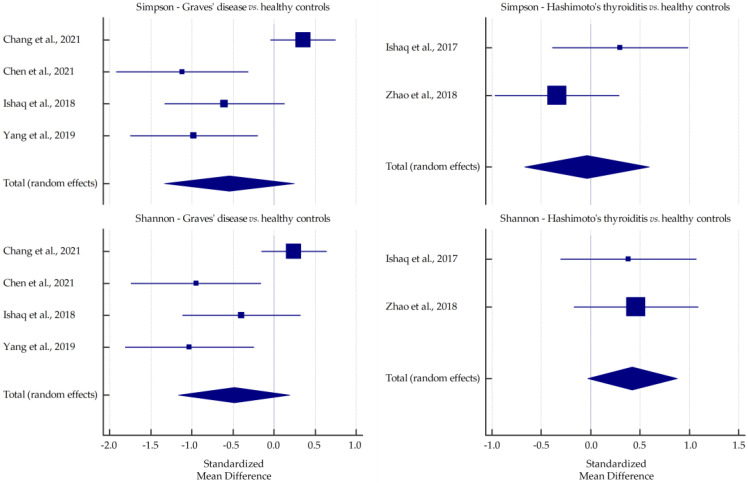
Pooled standardized mean differences in Simpson and Shannon indices, shown separately for Graves’ disease and Hashimoto’s thyroiditis [30,31,32,39,43,45].

Pooled standardized mean differences in richness (ACE and Chao1) and diversity (Simpson and Shannon) indices for GD and HT are plotted in Figure 3 and Figure 4. All values were lower in GD patients than in healthy subjects, especially Chao1 at the borderline of significance (*p*-value = 0.068). However, in HT patients, significantly higher mean values of ACE and Chao1 indices were observed than in healthy controls (*p*-value = 0.014 and *p*-value = 0.008, respectively) and the Shannon index was increased at the margin of significance (*p*-value = 0.068).

Significant differences in the relative abundance (at the phylum and genus levels) between the included studies (which reported *p*-values for comparisons) are presented in Table 3.

**Figure 5:**
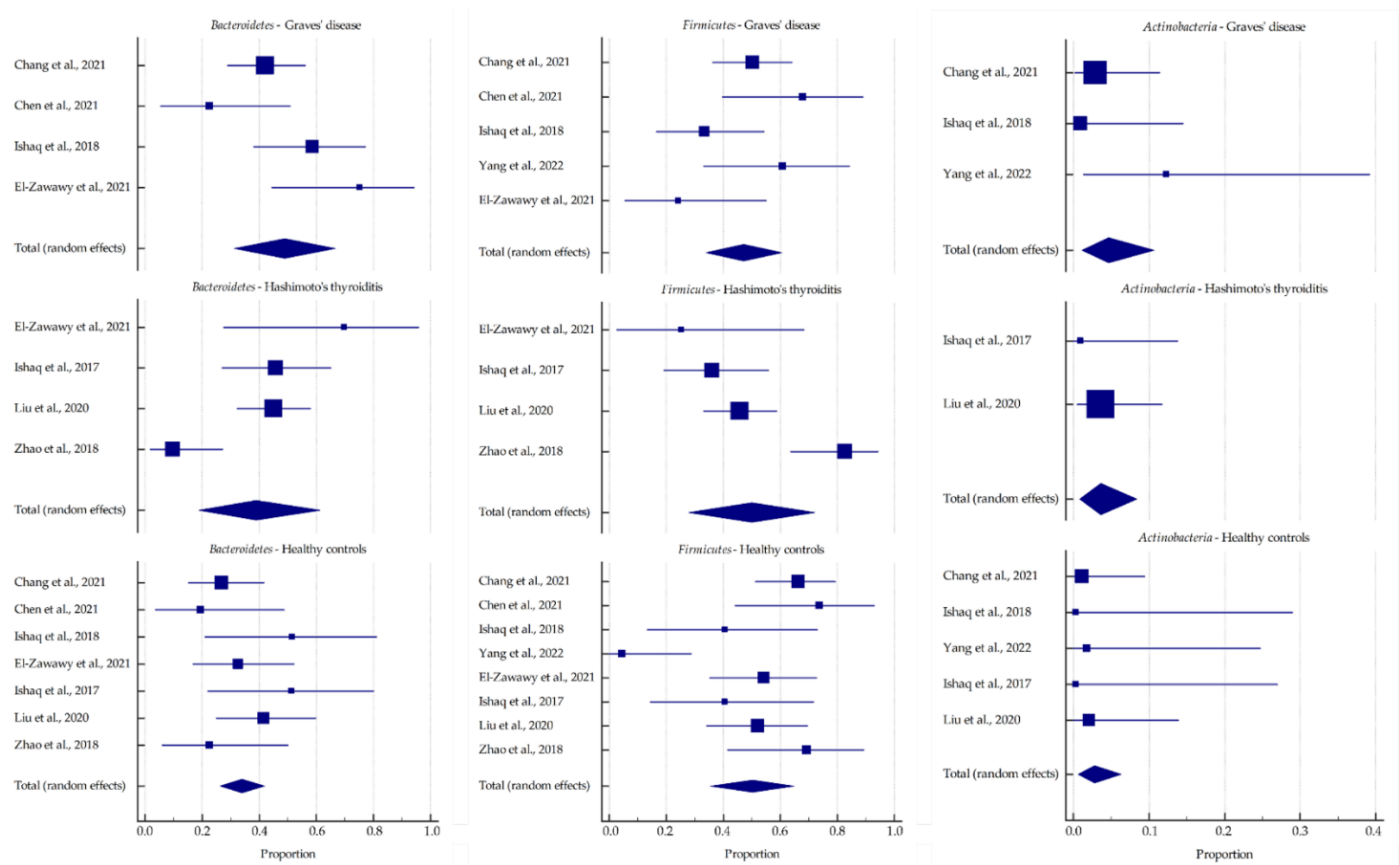
Forest plots representing the pooled relative abundance for the most common phyla in the included studies [30,31,32,38,41,43,44,45].

**Figure 6:**
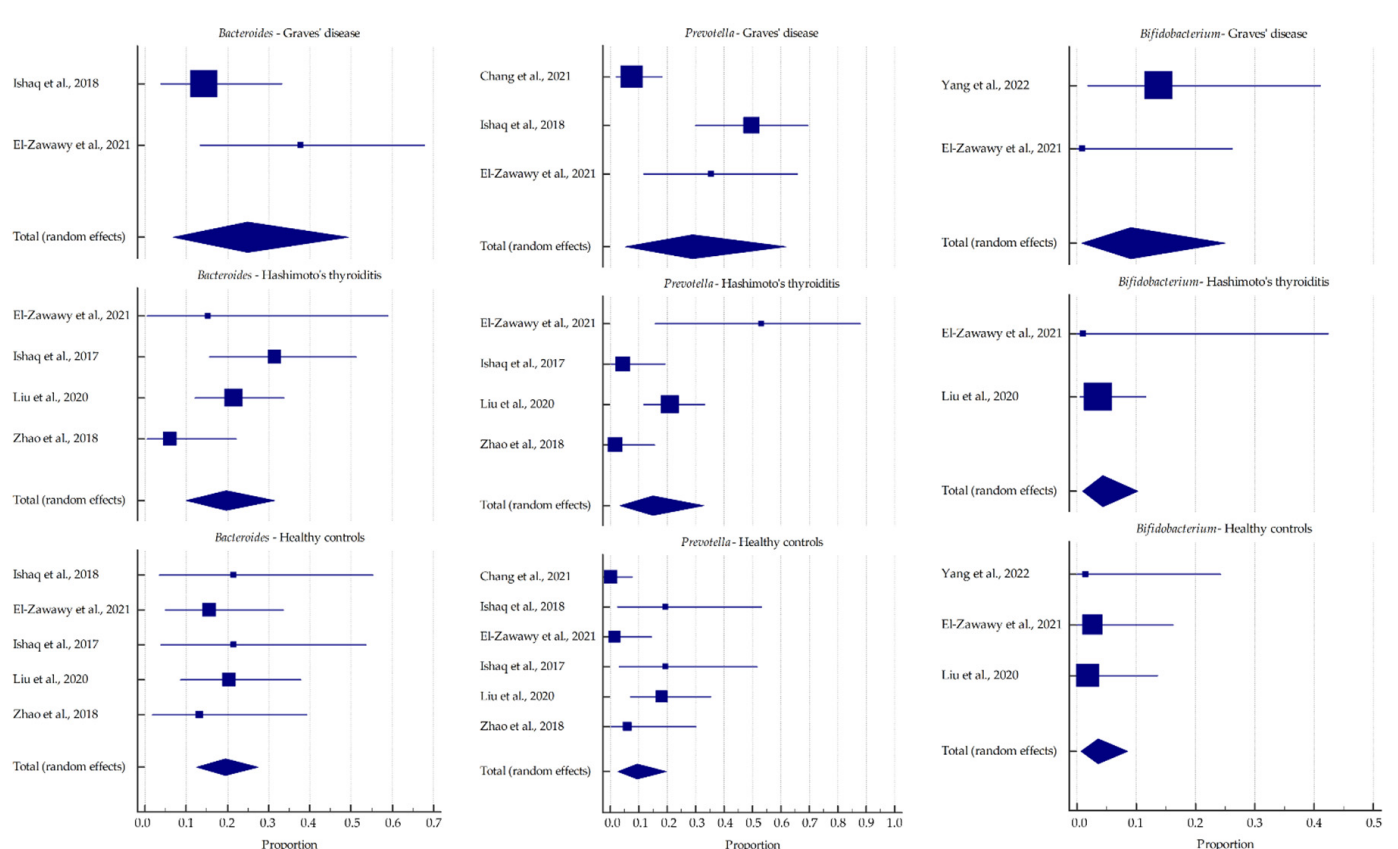
Forest plots representing the pooled relative abundance for the most common genera in the included studies [30,32,38,41,43,44,45].

The pooled relative abundance calculated for the most common phyla and genera from the included studies is represented in Figure 5 and Figure 6, respectively, and their values are presented in Table 4. In GD patients, a trend toward an increased abundance of *Bacteroidetes* and *Actinobacteria* was observed at the phylum level. This was reflected in a higher abundance of *Prevotella* and *Bifidobacterium* at the genus level. In summary, HT patients showed an abundance of the selected microbiota similar to those of healthy subjects.

In addition, significant correlations between microbiota alterations (at the phylum and genus levels) and the thyroid functional parameters determined, such as thyroid peroxidase antibody (TPOAb), TSH-receptor antibody (TRAb), thyroglobulin antibody (TGAb), and thyroid-stimulating hormone (TSH), are presented in Table 5.

## 3. Discussion

### 3.1. Microbiota Alpha-Diversity in AITD Patients

The diversity of the bacterial microbiota and the abundance of microbial species are characterized using specific alpha-diversity indices. Chao1 and ACE indices reflect the richness, whereas the Simpson index and the Shannon index reflect the community diversity (both richness and evenness) [30].

In all the studies included in this review, lower levels of richness indices (ACE and Chao1) were reported in GD patients than in healthy subjects. Statistically significant differences were reported in three studies [32,33,36]. For the Shannon index, significantly lower scores (including GO patients) were reported in six studies [31,33,34,35,36,37], and for the Simpson index, significantly lower scores were reported in three studies [31,34,36] and significantly higher scores were reported in one study [33]. One study reported strikingly contrasting results, reporting higher values (but not statistically significant) for all alpha-diversity indices in GD patients [30]. Despite this study, the meta-analysis showed a clear trend toward decreasing values of all indices in GD patients compared with healthy controls. It can be hypothesized that the lower diversity may be associated with the inflammatory response to the altered host immune function [33]. In addition, the decrease in microbial diversity may lead to a reduction in its functional capabilities, making it more susceptible to deleterious effects due to external disturbances [46]. Furthermore, this state is associated with several pathological conditions such as obesity, diabetes, inflammatory bowel diseases, polycystic ovary syndrome, and colorectal cancer [47,48,49,50,51]. Interestingly, Chen et al. [31] reported that microbiota diversity was significantly improved after 3–5 months of methimazole treatment.

However, in two studies, higher values of richness indices were reported in HT patients than in healthy subjects [43,45]. Similar findings were reported for the Simpson and the Shannon indices in these studies. The Shannon index was lower in HT patients than in healthy subjects in two studies [41,44]. The present meta-analysis confirmed increases in both richness indices and the Shannon index (as opposed to the Simpson index) in HT patients. This may be related to intestinal dysmotility and thus longer gastrointestinal transit time, predisposing to bacterial overgrowth in patients with hypothyroidism [52]. Although high microbiota diversity is generally associated with better health outcomes, it can also cause damaging effects such as increased protein breakdown and decreased polyphenol conversion, mucus secretion, and epithelial turnover [18].

Moreover, a Good’s coverage index of more than 99% was reported in the majority of the included studies, indicating that the current sequencing depth represented the true situation of fecal samples for gut microbiota. It should also be noted that the type of diet, eating habits, and geographical provenance can strongly influence gut microbiota diversity [53,54]. However, the effect of dietary factors was not considered in most of the studies, and only five studies excluded pure vegetarians [30,31,34,35,41]. Losasso et al. [55] reported a significantly higher richness in vegetarians than in omnivorous participants based on the Chao1 indices. However, these differences between omnivores and vegans or vegetarians were not confirmed in other studies [56,57]. Another potential confounding factor was cigarette smoking, one of the most important risk factors for GD and GO [58], since it was found to affect the composition of the gut microbiota [59,60,61].

### 3.2. Microbiota Relative Abundance in GD and HT Patients

In recent years, a possible role of the gut microbiome in the development and progression of diversity has been suggested, and its characteristics have been evaluated. Similar to the alpha-diversity analysis results, the intestinal microbiota composition at the phylum and genus levels was markedly different between GD patients and healthy controls. *Firmicutes* and *Bacteroidetes* phyla are the predominant components of the human gut microbiota, together comprising 90% of the total community. The relationship between these two phyla, expressed as the *Firmicutes*/*Bacteroidetes* ratio (F/B ratio), is a relevant marker of gut dysbiosis and is associated with various pathological conditions [62,63]. In most of the included studies, the F/B ratio was lower in GD patients than in healthy individuals, suggesting that this index could contribute to the pathogenesis of GD. A similar relationship was observed in GO patients [34,35]. However, Yang et al. [39] reported a significantly higher proportion of *Firmicutes* and a significantly lower proportion of *Bacteroidetes* in GD patients than in controls. Interestingly, an increased F/B ratio is usually observed in obesity [47]. Therefore, the effect of thyroid hormones on the composition and functioning of the intestinal microbiota, in addition to the increased basal metabolic rate, may result in weight changes [33]. This meta-analysis reported that the abundance of *Bacteroidetes* was higher in AITD patients, but that of *Firmicutes* did not differ compared with healthy subjects.

At lower taxonomic levels, the abundance of the genera was much more variable in both GD patients and healthy controls. Compared with the healthy controls, a significantly higher abundance of *Bacteroides* was reported in GD patients in two studies [30,33]. However, Shi et al. [35] reported that *Bacteroides* was significantly less abundant in the intestinal microbiota of GD patients than in healthy controls. Similar findings were reported in a murine model of GD/GO, where the INDIGO European consortium identified several disease-associated taxa, including reduced *Bacteroides* [64]. The gut microbiota of the murine model of GD/GO was also manipulated by antibiotics, probiotics, and human fecal material transfer, which resulted in the onset and modulation of the disease, thus confirming the effect of gut microbiota on GO [65]. The same international research group later confirmed a lower abundance of *Bacteroides* and a higher abundance of *Actinobacteria* in GD and GO patients compared with healthy controls (data unpublished). *Bacteroides* ferments glucose and lactate to SCFAs other than butyrate, such as acetate, succinate, and propionate, resulting in the reduction in mucin synthesis, tight junctions, and increased intestinal permeability, also known as the leaky gut syndrome (LGS). Furthermore, this leads to the disruption of gut homeostasis and may be involved in the pathogenesis and exacerbation of autoimmune disorders [66]. Moreover, in GD patients, a significant increase in *Prevotella* was reported in five studies [30,32,33,37,41]. In chronic inflammatory diseases, *Prevotella* mediate mucosal inflammation, which leads to the systemic dissemination of inflammatory mediators and bacterial products. Species of this genus predominantly activate Toll-like receptor 2, thus inducing the secretion of Th17-polarizing cytokines such as interleukin-1β, interleukin-6, and interleukin-23, and promote neutrophil recruitment by stimulating interleukin-17 production [67]. Yan et al. [37] reported that *Prevotella* might also affect the therapeutic efficacy of drugs for GD. Similarly, a higher abundance of *Prevotella* was reported in GO patients [34].

In addition, a higher abundance of the phylum *Actinobacteria* was reported, compared with healthy subjects, especially for two genera. Interestingly, the role of *Bifidobacterium* in immunopathogenesis is not clear since they may be protective or progressive in autoimmune diseases depending on the species [68]. For example, *Bifidobacterium bifidum* induces interleukin-17 secretion, promoting Th17 polarization, which is associated with autoimmune diseases [69]. Furthermore, the increased abundance of *Collinsella* is associated with an excessive interleukin-17 release and the altered permeability of the intestinal mucosa [70].

However, the relative abundance of the gut microbiota in HT patients was similar to that of healthy subjects, showing the reverse trend of alterations compared with GD patients. *Blautia*, the genus representative of the *Firmicutes* phylum, is an example of inverse dependencies between GD and HT. It is hypothesized that these commensal bacteria can mediate beneficial anti-inflammatory effects [71]. In addition, the abundance of *Blautia* was found to be significantly negatively correlated with visceral fat accumulation regardless of gender [72].

It is important to emphasize that similar to diversity, the relative abundance of the gut microbiota is also influenced by the type of diet. Previous studies reported that the Mediterranean diet was associated with the abundance of fiber-degrading bacterial genera such as *Bifidobacterium*, *Prevotella*, and *Roseburia*, and the suppression of *Streptococcus* and *Ruminococcus* [57,73]. However, the Western diet with higher fast-food consumption was characterized by decreased levels of *Lactobacillus* and *Faecalibacterium* [73,74].

### 3.3. Correlations between Microbiota Alterations and Thyroid Functional Parameters

The role of microbiota in the development of AITDs could be more clearly understood by investigating the relationships between changes (both functional and immunological) in its composition and thyroid functional parameters. Among the thyroid functional parameters determined, the most significant correlations were reported for TPOAb. Similarly, TSH and TRAb levels were often correlated with microbiota alterations. Only a few bacteria were correlated with the TGAb level.

However, the exact directions of these correlations are difficult to determine. At the phylum level, *Bacteroidetes* correlated negatively with the TSH level and positively with TPOAb and TRAb levels [30,36,41]. On the other hand, *Proteobacteria* and *Synergistetes* showed strikingly contrasting relationships [31,36].

At the genus level, especially in the phylum *Firmicutes*, a significant discrepancy in results was observed. *Veillonella* and *Streptococcus* correlated negatively with the TSH level and positively with TPOAb and TRAb levels, the latter genus correlated also with TGAb [36,45]. Moreover, *Bifidobacterium* showed the same findings [38]. However, *Faecalibacterium* and *Phascolarctobacterium* strains, as well as *Bacteroides* strains, showed contrasting correlations [30,31,33,36,40,45].

These findings confirm the significant correlations between some of the gut bacteria and thyroid parameters, indicating that microbiota alterations could be closely related to the development and progression of AITDs. *Veillonella* and *Streptococcus* are responsible for the development of oral diseases such as periodontitis and caries [75]. Both these genera interact metabolically and induce cytokine secretion by dendritic cells, resulting in an excessive immune response that may disrupt thyroid autoimmunity [76]. Moreover, *Lactobacillus* and *Bifidobacterium* strains have amino acid sequences familiar with TG and TPO, which can selectively bind with autoantibodies, triggering AITDs via molecular simulation mechanisms [77].

In contrast, *Faecalibacterium* is considered a protective factor in autoimmune processes, and its lower abundance stimulates the development of gastrointestinal disorders such as inflammatory bowel diseases and colorectal cancer [78]. Moreover, it is associated with a significant decrease in thyroid-stimulating immunoglobulin antibodies [40]. Similarly, a lower abundance of *Phascolarctobacterium* may lead to the altered production of SCFAs and thus an imbalance in immune homeostasis, increasing the host’s susceptibility to digestive and metabolic diseases [79]. At the phylum level, a lower abundance of *Synergistetes* may be involved in the balance of Th17/Treg differentiation, affecting the synthesis and secretion of autoantibodies in patients with autoimmune diseases [80].

Furthermore, the use of antithyroid drugs (ATD) might affect the gut microbiota composition. Little is known in this regard, except for in vitro studies that reported minor effects of ATD on 40 selected bacterial strains, even though other types of drugs were also able to inhibit the growth of one or more bacterial strains [81].

Moreover, gender, as well as the levels of sex hormones, could also affect the composition of intestinal microbes [82]. In women, subclinical thyroid abnormalities are more common and challenging to resolve [83]. The occurrence of subclinical hypothyroidism (SCH) may be associated with small intestinal bacterial overgrowth (SIBO). Wang et al. [84] reported a higher TPOAb-positive rate in SIBO-positive patients compared with SIBO-negative patients. In another study on pregnant women with SCH, differences in the gut microbiota composition and metabolic function were observed between TPOAb-positive and TPOAb-negative patients [85].

### 3.4. Study Limitations

The limitations of this systematic review include, in particular, the limitations of the selected studies. Sample sizes of these studies were relatively small, sometimes unmatched by age or gender in the case of control subjects. The majority of the selected studies were conducted in Asia, and one study each was conducted in Europe, Africa, and South America. Unfortunately, it was not possible to include all studies in the meta-analysis as complete data on diversity indices and relative abundance of the selected phyla and genera were not available (but only in diagrams with inaccurate scales). Only one study explicitly described GO patients, even though data from other independent groups are becoming available. It should be noted that all these studies used fecal samples to determine microbiota alterations, but the oral microbiome was not investigated, which may be of interest for further studies.

## 4. Materials and Methods

### 4.1. Search Strategy and Data Extraction

A systematic review was conducted up to 23 May 2022, according to the Preferred Reporting Items for Systematic Reviews and Meta-Analyses (PRISMA) statement guidelines [86], using the databases PubMed, Scopus, and Web of Science. The search queries included the following:

For PubMed: (((thyroid OR Graves) AND (orbitopathy OR ophthalmopathy)) OR ((Graves OR Hashimoto) AND (disease OR thyroiditis))) AND (microbiome OR microbiota OR microflora)

For Scopus: TITLE-ABS-KEY((((thyroid OR Graves) AND (orbitopathy OR ophthalmopathy)) OR ((Graves OR Hashimoto) AND (disease OR thyroiditis))) AND (microbiome OR microbiota OR microflora))

For Web of Science: TS = ((((thyroid OR Graves) AND (orbitopathy OR ophthalmopathy)) OR ((Graves OR Hashimoto) AND (disease OR thyroiditis))) AND (microbiome OR microbiota OR microflora)).

The results were filtered by the publication date (i.e., studies published after 2000). The title, abstract, and full text of the results were screened by two independent investigators. Studies included in this review matched all the predefined criteria according to PICOS (“Population”, “Intervention”, “Comparison”, “Outcomes”, and “Study design”), as shown in Table 6. A detailed search flowchart is presented in the Section 2. The study protocol was registered in the international prospective register of systematic reviews PROSPERO (CRD42022335984).

The results of the meta-analysis were presented in forest plots using MedCalc Statistical Software, version 19.5.3 (MedCalc Software Ltd., Ostend, Belgium). The meta-analysis was performed using the subgroups GD and HT. Pooled standardized mean differences for diversity indices were calculated as continuous variables and pooled proportions for the relative abundance at the phylum and genus levels.

### 4.2. Quality Assessment and Critical Appraisal for the Systematic Review of the Included Studies

**Figure 7:**
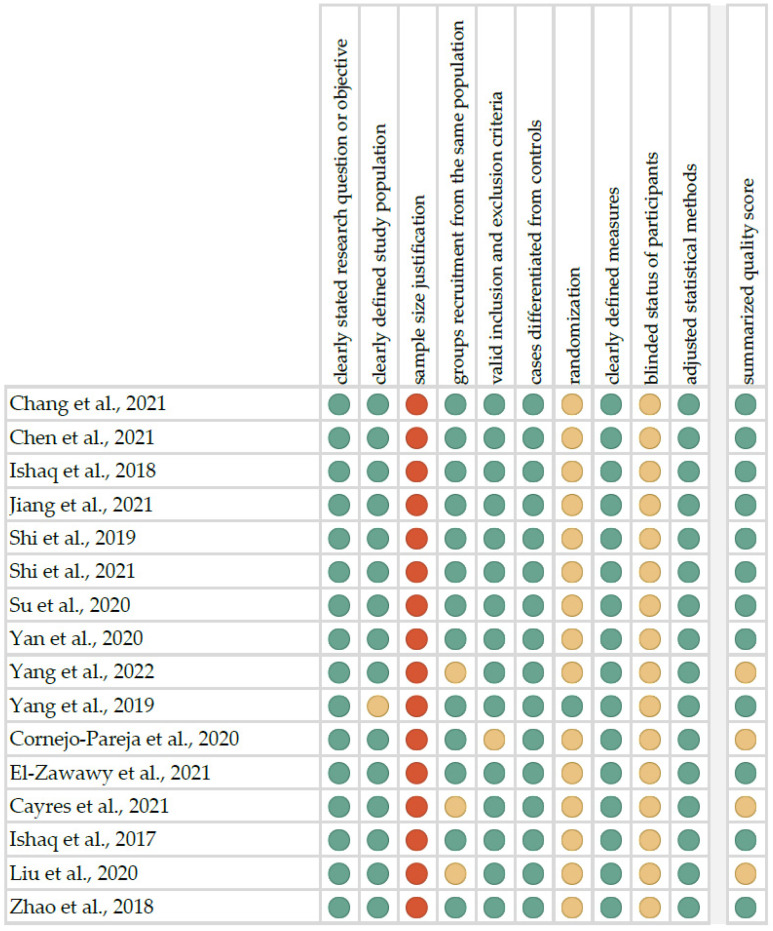
Quality assessment, including the main potential risk of bias (risk level: green—low, yellow—unspecified, red—high; quality score: green—good, yellow—intermediate, red—poor) [30,31,32,33,34,35,36,37,38,39,40,41,42,43,44,45].

The risk of bias in each of the selected studies was assessed using the “Study Quality Assessment Tool” issued by the National Heart, Lung, and Blood Institute within the National Institute of Health [87]. This questionnaire was answered by the two independent investigators, and any disagreements were resolved by discussion between them. The summarized quality assessment for the individual studies is represented in Figure 7. Critical appraisal was summarized by adding the points for each criterion of potential risk (points: 1—low, 0.5—unspecified, 0—high). Twelve studies (75.0%) were classified as having “good” quality (≥80% total score) and four (25.0%) as having “intermediate” quality (≥60% total score). The level of evidence was evaluated using the classification of the Oxford Centre for Evidence-Based Medicine levels for diagnosis [88]. All of the included studies showed the third or fourth level of evidence (in this 5-grade scale).

## 5. Conclusions

The findings of this systematic review showed that significant alterations in the diversity and composition of the intestinal microbiota can be observed in both GD and HT patients. Compared with healthy subjects, higher diversity indices were observed in HT patients, whereas lower values were observed in GD patients. Moreover, a higher relative abundance of *Bacteroidetes* and *Actinobacteria* was observed in GD patients. Changes in the composition of microbiota are most commonly correlated with TPOAb levels. Further studies are required to confirm these findings.

## Figures and Tables

**Table 1:** General characteristics of the included studies.

Author, Year	Setting	Study Group (F/M; Age)	Control Group (F/M; Age)	AITD Diagnosis	Inclusion Criteria	Exclusion Criteria	Thyroid Parameters Determined	Treatment
Chang et al., 2021 [30]	Taiwan	55 (35/20); 45.09 ± 12.08	48 (30/18); 42.60 ± 9.78	GD	Previously diagnosed with GD	Pregnancy, gastrointestinal disorders, gout, stroke, cancer, autoimmune diseases, history of gastrointestinal surgery, use of antibiotics, probiotics, prebiotics or symbiotics (<2 months), hormonal medication, Chinese herbal medicine (<3 months), pure vegetarians	TSH, FT4, TPOAb	PTU, MMI, CBZ
Chen et al., 2021 [31]	China	15 (8/7); 28.87 ± 6.79	14 (8/6); 27.29 ± 5.73	GD	Previously diagnosed GD	Untreated, transient hyperthyroidism, autoimmune diseases, long-term hormone treatment, diabetes, metabolic diseases, constipation, chronic diarrhea, inflammatory bowel disease, cancer, familial genetic disease, severe dysfunction of multiple organs, use of antibiotics, microbiological preparations (<1 month), vegetarians, pregnancy, lactation, history of gastrointestinal surgery, addiction to alcohol or drugs	TSH, FT3, FT4, T3, T4, TGAb, TPOAb, TRAb	MMI
Ishaq et al., 2018 [32]	China	27 (17/10); range: 35–50	11 (7/4); age-matched	GD	Previously diagnosed GD	GD treatment (<6 months), gastrointestinal diseases, use of prebiotics, probiotics, or antibiotics (<2 months)	TSH, FT3, FT4, T3, T4, TGAb, TPOAb, TRAb	No treatment (<6 months)
Jiang et al., 2021 [33]	China	45 (33/12); 37 (range: 16–65)	59 (37/22); 43 (range: 22–71)	GD	Previously diagnosed GD	Malignancies, gastrointestinal diseases, endocrine system diseases, use of antibiotics, probiotics, or prebiotics (<1 month)	TSH, FT3, FT4, T3, T4, TGAb, TPOAb, TRAb, TMAb	Untreated
Shi et al., 2019 [34]	China	33 (16/17); 46.0 ± 11.71	32 (16/16); 43.4 ± 9.7	GO	Diagnosed with Graves’ orbitopathy, CAS ≥ 3/7, and NOSPECS score ≥IV	Age < 18 or >65 years, use of antibiotics or probiotics (<4 weeks), use of hormonal medication, Chinese herbal medicine (<3 months), chronic diarrhea or constipation, inflammatory bowel disease, acute infections, diabetes, stroke, heart diseases, renal or hepatic dysfunction, cancer, autoimmune diseases, gastrointestinal surgery, pure vegetarians, pregnancy, lactation, alcohol or substance addiction	TSH, FT3, FT4, T3, T4, TGAb, TPOAb, TRAb	MMI
Shi et al., 2021 [35]	China	GO: 33 (16/17); 46.0 ± 11.7; GD: 30 (20/10); 45.0 ± 12.8	32 (16/16); 43.4 ± 9.7	GD/GO	Diagnosed with GD/GO	Age < 18 or >65 years, use of antibiotics or probiotics (<4 weeks), use of hormonal medication, Chinese herbal medicine (<3 months), chronic diarrhea or constipation, inflammatory bowel disease, acute infections, diabetes, stroke, heart diseases, renal or hepatic dysfunction, cancer, autoimmune diseases, gastrointestinal surgery, pure vegetarians, pregnancy, lactation, alcohol or substance addiction	TSH, FT3, FT4, T3, T4, TGAb, TPOAb, TRAb	MMI
Su et al., 2020 [36]	China	58 (35/23); 42.07 ± 10.22	63 (35/28); 43.86 ± 9.20	GD	Diagnosed with GD	Pregnancy, smoking, alcohol addiction, diarrhea, hypertension, diabetes, lipid dysregulation, BMI > 27, use of antibiotics, probiotics, prebiotics, symbiotics, hormonal medication, laxatives, proton pump inhibitors, insulin sensitizers, Chinese herbal medicine (<3 months), autoimmune diseases, malignancy, history of gastrointestinal surgery	TSH, FT3, FT4, TGAb, TPOAb	Untreated
Yan et al., 2020 [37]	China	39 (28/11); 37.49 ± 12.95	17 (11/6); 33.42 ± 9.13	GD	Diagnosed with GD	Pregnancy, smoking, alcohol addiction, use of antibiotics, hormonal medication, Chinese herbal medicine (3 months), use of medicine for the treatment of GD (<6 months), gastrointestinal diseases	TSH, T3, T4, TGAb, TPOAb, TRAb	No treatment (<6 months)
Yang et al., 2022 [38]	China	191 (116/75); mean: 45.8	30 (NR); NR	GD	Newly diagnosed with GD, without receiving any treatment, participating voluntarily with compliance, and written informed consent	Use of probiotics, prebiotics, antibiotics, Chinese herbal medicine (<1 month), complications of infection-associated diseases or other diseases (<1 month), chronic stress	TSH, FT3, FT4, TPOAb, TGAb TRAb	Untreated
Yang et al., 2019 [39]	China	15 (NR); range: 46–55	15 (age- and sex-matched)	GD	Diagnosed with GD	Osteoporosis, pregnancy, autoimmune diseases, infectious diseases, use of antibiotics, probiotics, metformin, acarbose, herbal preparations, use of any antithyroid treatment, chronic stress	NR	NR
Cornejo-Pareja et al., 2020 [40]	Spain	GD: 9 (7/2); 46.2 ± 8.6; HT: 9 (9/0); 40.3 ± 9.6	11 (7/4); 48.8 ± 6.2	GD and HT	Diagnosed with GD or HT	Pregnancy, diabetes, autoimmune diseases, gastrointestinal disorders, extreme diets, use of antibiotics, probiotics (<3 months), nonacceptance of informed consent	TSH, FT3, FT4, TPOAb, TSIAb	GD: CBZ; HT: LT4
El-Zawawy et al., 2021 [41]	Egypt	GD: 13 (4/9); 38.2; HT: 7 (6/1); 39.4	30 (17/13); 39.7 ± 10.9	GD and HT	Newly diagnosed and uncontrolled AITD (GD or HT)	Malignancy, recent gastrointestinal surgery (<6 months), recent hospitalization, use of antibiotics, nonsteroidal anti-inflammatory drugs, corticosteroids (<3 months), infectious diarrhea, other comorbidities, autoimmune diseases, pregnancy, severe burn, sepsis, pure vegetarians, smoking, alcohol or substance addiction, unable to give consent as mentally challenged, children	TSH, FT3, FT4, TPOAb, TRAb	NR
Cayres et al., 2021 [42]	Brazil	40 (36/4); 48.9 ± 13.3	53 (NR); 45.6 ± 16.7	HT	Diagnosed with HT	Use of anti-inflammatories, immunosuppressant drugs, antibiotics, vaccination (<30 days), gastrointestinal surgeries, inflammatory bowel diseases, chronic diarrhea	TSH, FT4, TPOAb, TGAb	LT4
Ishaq et al., 2017 [43]	China	29 (20/9); range: 40–60	12 (8/4); range: 40–60	HT	Diagnosed with HT	Gastrointestinal diseases, use of antibiotics, probiotics, and prebiotics (<60 days)	TSH, T3, T4, TPOAb, TGAb	NR
Liu et al., 2020 [44]	China	HTE: 45 (45/0); 34.6 ± 1.0 HTH: 18 (18/0); 36.3 ± 2.1	34 (34/0); 29.6 ± 0.6	HT	Diagnosed with HT	Autoimmune diseases, thyroid surgeries, pregnancy, use of antibiotics (<1 week)	TSH, FT3, FT4, T3, T4, TPOAb, TGAb	LT4
Zhao et al., 2018 [45]	China	Exploration cohort: 28 (25/3); 44.29 ± 12.25; validation cohort: 22 (19/3); 45.82 ± 10.7	Exploration cohort: 16 (14/2); 44.63 ± 10.33; validation cohort: 11 (9/2); 43.73 ± 10.95	HT	Diagnosed with HT, the presence of euthyroidism	Pregnancy, lactation, smoking, alcohol addiction, hypertension, diabetes, lipid dysregulation, BMI > 27, use of antibiotics, probiotics, prebiotics, symbiotics, hormonal medication, laxatives, proton pump inhibitors, insulin sensitizers, Chinese herbal medicine (<3 months), autoimmune diseases, malignancy, history of gastrointestinal surgery	TSH, FT3, FT4, TPOAb, TGAb	NR

Legend: NR, not reported; F, females; M, males; AITD, autoimmune thyroid disease; GD, Graves’ disease; GO, Graves’ orbitopathy; HT, Hashimoto’s thyroiditis; HTH, Hashimoto’s thyroiditis with hypothyroidism; HTE, Hashimoto’s thyroiditis with euthyroidism; BMI, body mass index; TPOAb, thyroid peroxidase antibody; TRAb, TSH-receptor antibody; TGAb, thyroglobulin antibody; TSH, thyroid-stimulating hormone; TSIAb, thyroid-stimulating immunoglobulin antibody; TMAb, thyroid microsomal antibody; FT3, free triiodothyronine; FT4, free thyroxine; T3, triiodothyronine; T4, thyroxine; PTU, propylthiouracil; MMI, methimazole; CBZ, carbimazole; LT4, levothyroxine; CAS, clinical activity score.

**Table 2:** Detailed characteristics of the included studies considering microbiological analysis and microbiota alterations.

Study	AITD Diagnosis	Type of Laboratory Material	Methods of Microbiological Analysis	Altered Microbiota Composition	Richness		Diversity	
					ACE	Chao1	Simpson	Shannon
Chang et al., 2021 [30]	GD	Fecal samples collected in a clean container, aliquoted, immediately frozen, and stored at −80 °C	16S rRNA gene sequencing	up: *Bacteroidetes*, *Actinobacteria*/*Bacteroides*, *Prevotella_9*; down: *Firmicutes*/*Faecalibacterium*, *Lachnospiraceae_NK4A136_group*	↑	↑	↑	↑
Chen et al., 2021 [31]	GD	Fecal samples collected on dry and clean paper, placed in sterile containers, transported at <4 °C, divided into portions, frozen, and stored at −80 °C	16S rDNA gene sequencing	up: *Lactobacillus*, *Veillonella*, *Streptococcus*; down: *Proteobacteria*, *Synergistetes*	-	↓	↓ *	↓ *
Ishaq et al., 2018 [32]	GD	Fecal samples collected in an icebox, transported within 1 h of defecation, and stored at −80 °C	16S rRNA gene sequencing	up: *Prevotella_9*, *Haemophilus*; down: *Alistipes*, *Faecalibacterium*, *Dialister*, *Bifidobacterium*, *Lactobacillus*	↓ *	↓ *	↓	↓
Jiang et al., 2021 [33]	GD	Fecal samples collected and stored at −80 °C	16S rRNA gene sequencing	up: *Bacteroidetes*/*Bacteroides*, *Lactobacillus*; down: *Firmicutes*/*Blautia*, *Eubacterium_hallii_group*, *Anaerostipes*, *Collinsella*, *Dorea*, *unclassified_f_Peptostreptococcaceae*, *Ruminococcus_torques_group*	↓ *	↓ *	↑ *	↓ *
Shi et al., 2019 [34]	GO	Fecal samples (2.5 g) collected in tubes prefilled with fecal DNA stabilizer and stored at −80 °C	16S rRNA gene sequencing	up: *Bacteroidetes*/*unidentified_Prevotellaceae*; down: *Firmicutes*/*Blautia*, *Fusicatenibacter*, *Butyricicoccus*, *Anaerostipes*, *Collinsella*	ns	ns	↓ *	↓ *
Shi et al., 2021 [35]	GD/GO	Fecal samples (2.5 g) collected in tubes prefilled with fecal DNA stabilizer and stored at −80 °C	16S rRNA gene sequencing	GO vs. GD: up: *Subdoligranulum*, *Bilophila*; down: *Deinococcus-Thermus*, *Chloroflexi*/*Blautia*, *Anaerostipes*, *Dorea*, *Butyricicoccus*, *Romboutsia*, *Fusicatenibacter*, *unidentified_Lachnospiraceae*, *unidentified_Clostridiales*, *Collinsella*, *Intestinibacter*, *Phascolarctobacterium*	ns	ns	-	↓ *
Su et al., 2020 [36]	GD	Fecal samples stored at −80 °C after liquid nitrogen freezing	16S rRNA gene sequencing	up: *Spirochaetae*, *Saccharibacteria*, *Bacteroidetes*; down: *Firmicutes*, *Proteobacteria*, *Synergistetes*, *Tenericutes*, *Verrucomicrobia*	↓ *	↓ *	↓ *	↓ *
Yan et al., 2020 [37]	GD	Fecal samples stored at −80 °C	16S rRNA gene sequencing	up: *Bacilli*, *Lactobacillales*, *Prevotella*, *Megamonas*, *Veillonella*; down: *Ruminococcus*, *Rikenellaceae*, *Alistipes*	-	ns	ns	↓ *
Yang et al., 2022 [38]	GD	Fecal samples (10 g) immediately stored in sterile iceboxes and stored at −80 °C	16S rRNA gene sequencing	up: *Actinobacteria*/*Bifidobacterium*, *Collinsella*, *Pediococcus*; down: *Firmicutes*/*Roseburia*, *Dialister*	ns	ns	ns	ns
Yang et al., 2019 [39]	GD	Fecal samples (2 g) stored at −80 °C	16S rRNA gene sequencing	up: *Firmicutes*, *Proteobacteria*, *Actinobacillus*/*Oribacterium*, *Mogibacterium*, *Lactobacillus*, *Aggregatibacter*; down: *Bacteroidetes*	↓	↓	↓	↓
Cornejo-Pareja et al., 2020 [40]	GD	Fecal samples immediately refrigerated and stored at −80 °C	16S rRNA gene sequencing	up: *Fusobacterium*, *Sutterella*; down: *Faecalibacterium*	-	-	-	ns
	HT			up: *Victivallaceae*	-	-	-	ns
El-Zawawy et al., 2021 [41]	GD	Fecal samples kept at −20 °C upon defecation at home and stored at −80 °C	16S rRNA gene sequencing	up: *Bacteroidetes*/*Prevotella*; down: *Firmicutes*	-	-	-	↓
	HT				-	-	-	↓
Cayres et al., 2021 [42]	HT	Fecal samples (200 mg)	16S rRNA gene sequencing	up: *Bacteroides*; down: *Bifidobacterium*	-	-	-	-
Ishaq et al., 2017 [43]	HT	Fecal samples collected in a sterile cup, transported within 4 h of defecation, and stored at −80 °C	16S rRNA gene sequencing	down: *Bifidobacterium*, *Lactobacillus*, *Dialister*	↑ *	↑ *	↑	↑
Liu et al., 2020 [44]	HT	Fecal samples collected in tubes prefilled with fecal DNA stabilizer and stored at −80 °C	16S rRNA gene sequencing	HTH: up: *Phascolarctobacterium*; HTE: up: *Lachnospiraceae_incertae_sedis*, *Lactonifactor*, *Alistipes*, *Subdoligranulum*	-	-	-	↓ *
Zhao et al., 2018 [45]	HT	Fecal samples immediately divided into aliquots, frozen on dry ice, and stored at −80 °C	16S rRNA gene sequencing	up: *Firmicutes*/*Blautia*, *Roseburia*, *Ruminococcus_torques_group*, *Romboutsia*, *Dorea*, *Fusicatenibacter*, *Eubacterium_hallii_group*; down: *Bacteroidetes*/*Faecalibacterium*, *Bacteroides*, *Prevotella_9*, *Lachnoclostridium*	↑	↑	↓	↑

Legend: AITD, autoimmune thyroid disease; GD, Graves’ disease; GO, Graves’ orbitopathy; HT, Hashimoto’s thyroiditis; -, not reported; ns, nonsignificant difference; *, significant difference; ↓, lowered value; ↑, increased value; ACE, abundance-based coverage estimator; HTH, Hashimoto’s thyroiditis with hypothyroidism; HTE, Hashimoto’s thyroiditis with euthyroidism.

**Table 3:** Relative abundance of significantly altered microbiota in autoimmune thyroid disease patients.

Study	AITD Diagnosis	Bacterial Phylum	Bacterial Genus	Relative Abundance		
				Study	Control	*p*-Value
Chang et al., 2021 [30]	GD	*Bacteroidetes*		0.4210	0.2690	<0.01
		*Actinobacteria*		0.0288	0.0111	<0.01
		*Firmicutes*		0.5020	0.6630	<0.01
			*Collinsella*	0.0052	0.0014	<0.01
			*Parabacteroides*	0.0110	0.0009	<0.01
			*Prevotella_9*	0.0767	0.0017	<0.01
Chen et al., 2021 [31]	GD	*Synergistetes*		0.000012	0.0024	0.028
			*Veillonella*	0.0172	0.0006	0.039
Ishaq et al., 2018 [32]	GD		*Prevotella_9*	0.4970	0.1952	0.034
			*Haemophilus*	0.1358	0.0099	0.049
			*Dialister*	0.0110	0.0445	0.047
			*Alistipes*	0.0180	0.0474	0.025
			*Faecalibacterium*	0.0289	0.0562	0.014
Yang et al., 2022 [38]	GD	*Actinobacteria*		0.1229	0.0175	0.003
		*TM7*		0.0001	0.00001	0.011
		*Firmicutes*		0.6088	0.7522	0.044
		*Cyanobacteria*		0.0002	0.00003	0.050
El-Zawawy et al., 2021 [41]	GD and HT	*Bacteroidetes*		0.738	0.326	<0.001
		*Firmicutes*		0.248	0.543	<0.001
			*Prevotella*	0.4000	0.0161	0.006
Ishaq et al., 2017 [43]	HT		*Dialister*	0.0091	0.0446	0.029
Zhao et al., 2018 [45]	HT	*Firmicutes*		0.826	0.691	<0.001
		*Bacteroidetes*		0.099	0.227	<0.001
			*Faecalibacterium*	0.0987	0.1510	0.004
			*Bacteroides*	0.0613	0.1330	<0.001
			*Prevotella_9*	0.0183	0.0601	<0.001
			*Blautia*	0.0977	0.0586	<0.001
			*Roseburia*	0.0398	0.0312	0.010
			*Lachnoclostridium*	0.0241	0.0285	0.013
			*Ruminococcus_torques_group*	0.0306	0.0200	0.002
			*Romboutsia*	0.0235	0.0146	0.006
			*Dorea*	0.0200	0.0138	0.006
			*Fusicatenibacter*	0.0186	0.0100	<0.001
			*Eubacterium_hallii_group*	0.0258	0.0110	<0.001

Legend: AITD, autoimmune thyroid disease; GD, Graves’ disease; GO, Graves’ orbitopathy; HT, Hashimoto’s thyroiditis.

**Table 4:** Pooled relative abundance (%) for the most common microbiota in autoimmune thyroid disease patients.

Microbial Phylum/Genus	Graves’ Disease (A)			Hashimoto’s Thyroiditis (B)			Healthy Controls (C)			*p*-Value		
	Mean, %	−95% CI	+95% CI	Mean, %	−95% CI	+95% CI	Mean, %	−95% CI	+95% CI	A vs. B	A vs. C	B vs. C
***Bacteroidetes***	48.964	31.446	66.615	39.038	19.009	61.224	33.948	26.366	41.968	0.487	0.089	0.628
*Bacteroides*	24.806	6.786	49.378	19.629	10.027	31.489	19.498	12.611	27.466	0.653	0.560	0.985
*Prevotella*	28.911	5.242	61.787	14.965	3.338	32.884	9.451	2.638	19.853	0.360	0.127	0.513
***Firmicutes***	47.207	34.033	60.582	49.955	27.895	72.025	50.185	35.499	64.854	0.835	0.779	0.986
***Actinobacteria***	4.692	1.087	10.638	3.641	0.824	8.347	2.77	0.632	6.356	0.737	0.482	0.713
*Bifidobacterium*	9.027	0.759	24.955	4.354	0.894	10.238	3.54	0.656	8.593	0.390	0.274	0.794

Legend: CI, confidence interval.

**Table 5:** Significant correlations between microbiota alterations with the thyroid functional parameters determined.

	TPOAb		TRAb		TGAb		TSH	
	Positive	Negative	Positive	Negative	Positive	Negative	Positive	Negative
***Phyla***	*Firmicutes* [41], *Bacteroidetes* [30,36,41], *Actinobacteria* [30]	*Firmicutes* [30], *Proteobacteria* [36], *Synergistetes* [31,36]	*Firmicutes* [41], *Bacteroidetes* [41]	*Proteobacteria* [36], *Synergistetes* [31,36]	*Actinobacteria* [36]	*Synergistetes* [31]	*Firmicutes* [30,36], *Proteobacteria* [36], *Synergistetes* [31,36]	*Bacteroidetes* [30,36]
***Genera***	*Blautia* [33,45], *Lactobacillus* [31], *Streptococcus* [36,45], *Veillonella* [36], *Alistipes* [40], *Prevotella* [30,36,41], *Bifidobacterium* [38]	*Blautia* [36], *Faecalibacterium* [30,40,45], *Phascolarctobacterium* [36,45], *Alistipes* [36], *Bacteroides* [33,36,45], *Prevotella* [45]	*Lactobacillus* [31], *Streptococcus* [36], *Veillonella* [36], *Prevotella* [36], *Bifidobacterium* [38]	*Blautia* [36], *Lactobacillus* [36], *Phascolarctobacterium* [31,36], *Alistipes* [36], *Bacteroides* [36]	*Blautia* [45], *Streptococcus* [36,45], *Prevotella* [36], *Bifidobacterium* [38]	*Phascolarctobacterium* [36,45], *Bacteroides* [45], *Prevotella* [45]	*Blautia* [36], *Faecalibacterium* [30], *Lactobacillus* [36], *Phascolarctobacterium* [31,36], *Alistipes* [36], *Bacteroides* [36]	*Lactobacillus* [31], *Streptococcus* [36], *Veillonella* [36], *Prevotella* [30,36]

Legend: TPOAb, thyroid peroxidase antibody; TRAb, TSH-receptor antibody; TGAb, thyroglobulin antibody; TSH, thyroid-stimulating hormone.

**Table 6:** Inclusion and exclusion criteria according to the PICOS.

Parameter	Inclusion Criteria	Exclusion Criteria
Population	Patients with autoimmune thyroid diseases, including Graves’ disease and Hashimoto’s thyroiditis, aged 0–99 years, both sexes	Patients with other autoimmune diseases
Intervention	Not applicable	
Comparison	Not applicable	
Outcomes	Alterations in microbiota composition, including richness, diversity, and abundance indices	Alterations in microbiota composition without determined diversity indices
Study design	Case–control, cohort, and cross-sectional studies	Literature reviews, case reports, expert opinions, letters to the editor, conference reports
	Published after 2000	Not published in English

## Data Availability

Not applicable.
